# Bacterial Pneumonia among HIV-Infected Patients: Decreased Risk After Tobacco Smoking Cessation. ANRS CO3 Aquitaine Cohort, 2000–2007

**DOI:** 10.1371/journal.pone.0008896

**Published:** 2010-01-26

**Authors:** Antoine Bénard, Patrick Mercié, Ahmadou Alioum, Fabrice Bonnet, Estibaliz Lazaro, Michel Dupon, Didier Neau, François Dabis, Geneviève Chêne, the Groupe d'Epidémiologie Clinique du Sida en Aquitaine (GECSA)

**Affiliations:** 1 INSERM, U897, CIC-EC7, Bordeaux, France; 2 Université Victor Segalen Bordeaux 2, ISPED, Bordeaux, France; 3 CHU de Bordeaux, COREVIH Aquitaine, Bordeaux, France; University of Cape Town, South Africa

## Abstract

**Background:**

Bacterial pneumonia is still a substantial cause of morbidity and mortality in HIV-infected patients in the era of combination Antiretroviral Therapy. The benefit of tobacco withdrawal on the risk of bacterial pneumonia has not been quantified in such populations, exposed to other important risk factors such as HIV-related immunodeficiency. Our objective was to estimate the effect of tobacco smoking withdrawal on the risk of bacterial pneumonia among HIV-infected individuals.

**Methodology/Principal Findings:**

Patients of the ANRS CO3 Aquitaine Cohort with ≥ two visits during 2000–2007 and without bacterial pneumonia at the first visit were included. Former smokers were patients who stopped smoking since ≥ one year. We used Cox proportional hazards models adjusted on CD4+ lymphocytes (CD4), gender, age, HIV transmission category, antiretroviral therapy, cotrimoxazole prophylaxis, statin treatment, viral load and previous AIDS diagnosis. 135 cases of bacterial pneumonia were reported in 3336 patients, yielding an incidence of 12 ‰ patient-years. The adjusted hazard of bacterial pneumonia was lower in former smokers (Hazard Ratio (HR): 0.48; *P* = 0.02) and never smokers (HR: 0.50; *P* = 0.01) compared to current smokers. It was higher in patients with <200 CD4 cells/µL and in those with 200 to 349 CD4 cells/µL (HR: 2.98 and 1.98, respectively; both *P*<0.01), but not in those with 350 to 499 CD4 cells/µL (HR: 0.93; *P* = 0.79), compared to those with ≥500 CD4 cells/µL. The interaction between CD4 cell count and tobacco smoking status was not statistically significant.

**Conclusions/Significance:**

Smoking cessation dramatically reduces the risk of bacterial pneumonia, whatever the level of immunodeficiency. Smoking cessation interventions should become a key element of the clinical management of HIV-infected individuals.

## Introduction

Bacterial pneumonia is still a substantial cause of morbidity and mortality in HIV-infected patients in the era of combination Antiretroviral Therapy (cART). Indeed, around 10% of the causes of severe morbidity and 5% of the causes of death are related to a bacterial pneumonia in industrialized countries [1], [2]. In the general population, tobacco smoking is well-known as a major modifiable risk factor of respiratory tract infections [3]. Pulmonary infections in tobacco smokers are mediated by local inflammatory modifications and a significant depression in the percentage and absolute numbers of CD4+ and CD8+ lymphocytes [4]. Among HIV-infected patients, the prevalence of tobacco smoking is very high, generally reported around 50% in European HIV cohorts [5], [6], compared to 27% in the general population of comparable age and gender [7]. Recent studies have confirmed the effect of tobacco smoking on respiratory tract infections in HIV-infected patients [8], [9], [10], and promoted tobacco smoking withdrawal as a priority. However, the benefit of tobacco withdrawal on the risk of bacterial pneumonia has not been quantified in such populations, exposed to other important risk factors such as HIV-related immunodeficiency [11]. In addition, respiratory tract immunological modifications due to HIV infection might interact with the effect of tobacco smoking [12], [13], [14].

This study aimed at estimating the effect of tobacco smoking withdrawal on the risk of bacterial pneumonia among HIV-infected individuals in the era of cART, and its variation according to the level of immunodeficiency.

## Methods

### Ethics Statement

The ANRS CO3 Aquitaine cohort was granted ethics permission from the institutional review board of the ANRS (Agence nationale de recherched sur le sida et les hépatites virales). All patients included in the ANRS CO3 Aquitaine Cohort have signed an informed consent.

### Study Sample

All patients of the ANRS CO3 Aquitaine Cohort with at least two visits during the 2000–2007 period, free of bacterial pneumonia at first visit and with available data on tobacco consumption were included in the study. The ANRS CO3 Aquitaine Cohort enrols all HIV-infected in- or outpatients of the participating clinic wards of the Bordeaux University Hospital and five other public hospitals in Aquitaine, south-western France, since 1985 [15]. Tobacco smoking status is recorded at inclusion (history of tobacco smoking and current status) and at each visit (current status only) with clinical, biological and therapeutic data.

### Outcome Measures

Specific radiological signs including alveoli consolidation limited or not to a segment or lobe or diffuse bilateral reticulo-nodular pattern were a pre-requesite to define cases of bacterial pneumonia. Cases of pneumonia excluded tuberculosis and other mycobacteria. Bacterial pneumonia considered for this analysis were classified into two categories: (1) “confirmed” (clinical and specific radiological signs together with a bacteriological confirmation) or (2) “probable” (clinical and specific radiological signs together with a successful antibacterial treatment). Smoking status was defined as current smokers, never smokers and former smokers (i.e.: patients who had stopped smoking since at least one year) at each follow-up visit. Four categories of immunological status were defined based on CD4 count: <200 cells/µL, 200 to 349 cells/µL, 350 to 499 cells/µL and ≥500 cells/µL.

### Statistical Analysis

Time to a first episode of bacterial pneumonia was calculated as the difference between the date of the first visit in the 2000–2007 period and the date of diagnosis of bacterial pneumonia or the date of the last visit within the study. Tobacco smoking status was taken into account as a time-dependent variable as cessation attempts and relapses are frequent in HIV-infected populations (Encrenaz G et al. *Curr HIV Res.* 2009. In press). Explanatory variables included gender, age at first visit, HIV infection through intravenous drug use [IDU] and, as time-dependent variables, CD4 cell count, previous diagnosis of AIDS, plasma HIV RNA, cART (defined as a combination of at least three antiretrovirals), cotrimoxazole prophylaxis and statin treatment. Indeed, this latter variable has been reported as a protective factor in recent studies in patients with chronic conditions [16], [17]. Latest values were considered for time-dependent variables.

We estimated the incidence rate of bacterial pneumonia per 1000 patient-years with its corresponding 95% confidence interval (CI), assuming that time-dependent variables were constant between each measurement. Cox proportional hazards models including a term of interaction between CD4 cell count and tobacco smoking status were used. Statistical analyses were performed using SAS (version 9.1; SAS Institute, Cary, NC).

## Results

Out of 4365 patients enrolled in the ANRS CO3 Aquitaine Cohort seen at least once in 2000–2007, 3336 (76%) were included in this study. Eight hundred and fifty five (20%) patients had no data available on tobacco smoking status, 30 (1%) presented a bacterial pneumonia at the first visit and 144 (3%) had only one visit during the 2000–2007 period. The 1029 excluded patients did not differ significantly from the 3336 patients available for the analysis in terms of age (40 years old on average in both groups), HIV transmission categories (injecting drug users: 20% versus 21%; heterosexual: 36% versus 30%), proportion of cART-treated patients (64% versus 67%), plasma HIV RNA (54% versus 50% with ≥1000 copies/ml), mean CD4 count (449 versus 450 cells/µL) and proportion of patients with a previous AIDS diagnosis (21% in both groups). Excluded patients were less frequently male (68% as compared to 74% in the study sample, *P*<0.001). Baseline characteristics of the 3336 included patients are shown in table 1.

**Table 1:** Baseline characteristics of patients included in the analysis.

Category	Sub-category	N	Median	Interquartile Range	n	%	95% confidence interval
**Tobacco smoking status**		3336					
	Current smokers				1779	53.3	[51.6–55.0]
	Former smokers				411	12.3	[11.2–13.4]
	Never smokers				1146	34.3	[32.7–36.0]
**CD4+ lymphocyte count (cells/µL)**		3334	421.0	[254.0–603.0]			
	≥500				1238	37.1	[35.5–38.8]
	[350–499]				757	22.7	[21.3–24.2]
	200–349				742	22.3	[20.8–23.7]
	<200				597	17.9	[16.6–19.2]
**Gender**		3336					
	Men				2480	74.3	[72.9–75.8]
	Women				856	25.7	[24.2–27.1]
**Age** (years)		3336	39.6	[34.5–46.0]			
	<30				349	10.5	[9.4–11.5]
	[30–40]				1306	39.2	[37.5–40.8]
	[40–50]				1122	33.6	[32.0–35.3]
	[50–60]				397	11.9	[10.8–13.1]
	≥60				162	4.9	[4.2–5.6]
**Plasma HIV RNA (copies/ml)**		3303					
	≥1000				1643	49.7	[48.0–51.5]
	<1000				1660	50.3	[48.5–52.0]
**HIV transmission categories**		3336					
	Heterosexual				1015	30.4	[28.9–32.0]
	Homo- and bisexual				1305	39.1	[37.5–40.8]
	Injecting drug users				707	21.2	[19.8–22.6]
	Other				309	9.3	[8.3–10.3]
**CDC stage**		3336					
	A				1909	57.2	[55.6–58.9]
	B				728	21.8	[20.4–23.2]
	C				699	20.9	[19.6–22.3]
**cART**		3336					
	Yes				2231	66.9	[65.3–68.5]
	No				1105	33.1	[31.5–34.7]
**Cotrimoxazole prophylaxis**		3336					
	Yes				64	1.9	[1.4–2.4]
	No				3272	98.1	[97.6–98.6]
**Statin treatment**		3336					
	Yes				67	2.0	[1.6–2.5]
	No				3269	98.0	[97.5–98.4]

cART: combination Antiretroviral Therapy.

**ANRS CO3 Aquitaine Cohort, 2000–2007.**

Among the 1779 (53%) current smokers at inclusion, 277 (16%) stopped smoking for at least 1 year during follow-up and were considered former smokers. Among the 411 (12%) former smokers at inclusion, 164 (40%) relapsed during follow-up. Among the 1146 (34%) never smokers at inclusion, 41 (4%) did start smoking during follow-up. During a median follow-up of 3.3 years (inter-quartile range [IQR]: 1.6–5.1) and a median number of 12 visits (IQR: 6–21), 135 first episodes of bacterial pneumonia were reported. Of these, 51 (38%) required an hospitalization. Of the 104 episodes of bacterial pneumonia diagnosed in tobacco smokers, 38 (37%) required an hospitalization as compared to 50% in former and never smokers.

Overall, 104 (77%) of the bacterial pneumonia events were classified as probable. Of the 31 (23%) with documented bacteria (confirmed cases), *Streptococcus pneumoniae* (22 cases) was the most common. Other identified organisms were *Pseudomonas* (6 cases), *Staphylococcus aureus* (2 cases) and *Haemophilus influenzae* (1 case). Seventy eight (58%) pneumonia episodes were diagnosed between November and April, i.e influenza virus epidemics period.

The overall incidence of bacterial pneumonia was 12.0 per 1000 patient-years (CI: 9.9–14.0). It was 15.9 per 1000 patient-years (CI: 13.1–19.3) when patients were smoking, 7.9 per 1000 patient-years (CI: 4.2–14.9) in former smokers and 5.9 per 1000 patient-years (CI: 3.5–10.0) in never smokers. The incidence of bacterial pneumonia was 28.8 (CI: 16.7–49.8) and 16.5 (CI: 9.5–28.4) per 1000 patient-years among patients with <200 and those with 200 to 349 CD4 cells/µL, respectively. It was 7.7 per 1000 patient-years among patients with ≥350 CD4 cells/µL (table 2).

**Table 2:** Factors associated with the hazard of bacterial pneumonia among HIV-infected patients in the era of cART.

Category	Sub-category	Univariate analysis (3336 patients/135 events) Incidence rates (‰ patients-years)				Multivariate analysis 3303 patients/135 events		
		N events	Patients-years	Incidence	95% CI	Hazard Ratio	95% CI	*P*
**Tobacco smoking status**								
	Current smokers	104	6546	15.9	[13.1–19.3]	1		
	Former smokers	12	1513	7.9	[4.2–14.9]	0.48	[0.26–0.90]	0.02
	Never smokers	19	3228	5.9	[3.5–10.0]	0.50	[0.29–0.86]	0.01
**CD4+ lymphocyte count (cells/µL)**								
	≥500	39	4941	7.9	[5.8–10.8]	1		
	[350–499]	20	2717	7.4	[3.9–13.7]	0.93	[0.54–1.60]	0.79
	200–349	38	2309	16.5	[9.5–28.4]	1.98	[1.25–3.15]	0.004
	<200	38	1317	28.8	[16.7–49.8]	2.98	[1.80–4.94]	<0.001
**Gender**								
	Men	92	8433	10.9	[6.8–17.4]	1		
	Women	43	2853	15.1	[11.2–20.3]	1.56	[1.07–2.27]	0.02
**Age** (years)								
	<30	4	1084	3.7	[1.4–09.8]	1		
	[30–40]	64	4697	13.6	[3.3–55.7]	2.38	[0.86–6.62]	0.10
	[40–50]	47	3747	12.5	[3.0–51.6]	2.70	[0.96–7.61]	0.06
	[50–60]	13	1294	10.0	[2.3–44.5]	3.53	[1.13–10.98]	0.03
	≥60	7	464	15.1	[3.1–72.7]	8.30	[2.37–29.04]	<0.001
**Plasma HIV RNA (copies/ml)** *								
	<1000	72	7557	9.5	[7.1–11.1]	1		
	≥1000	63	3729	16.9	[11.2–25.4]	1.75	[1.19–2.56]	0.004
**HIV transmission categories**								
	Other	78	8778	8.9	[7.2–11.2]	1		
	Injecting drug users	57	2508	22.7	[15.1–34.1]	1.87	[1.27–2.73]	0.001
**Previous diagnosis of AIDS** *								
	No	90	8751	10.3	[8.4–12.6]	1		
	Yes	45	2535	17.8	[11.7–26.8]	1.20	[0.82–1.77]	0.35
**cART** *								
	No	30	2553	11.8	[8.2–16.8]	1		
	Yes	105	8733	12.0	[7.0–20.7]	0.89	[0.57–1.39]	0.60
**Cotrimoxazole prophylaxis** *								
	No	133	11141	11.9	[10.1–14.1]	1		
	Yes	2	145	13.8	[3.4–56.2]	0.76	[0.18–3.18]	0.71
**Statin treatment** *								
	No	131	10768	12.2	[10.3–14.4]	1		
	Yes	4	518	7.7	[2.8–21.2]	0.56	[0.20–1.54]	0.26

*Time-updated variables.

cART: combination Antiretroviral Therapy.

CI: Confidence Interval.

**ANRS CO3 Aquitaine Cohort, 2000–2007.**

In the multivariate analysis, the interaction between CD4 count and tobacco smoking status was not significant (*P* = 0.44 and 0.34 for the relation between current smokers and former and never smokers, respectively). The adjusted hazard of bacterial pneumonia was significantly lower in former smokers compared to current smokers (Hazard Ratio (HR): 0.48; CI: 0.26–0.90; *P* = 0.02). It was also significantly lower in never smokers compared to current smokers (HR: 0.50; CI: 0.29–0.86; *P* = 0.01). The hazard of bacterial pneumonia was higher when CD4 count was <200 cells/µL (HR: 2.98; CI: 1.80–4.94; *P*<0.001) or between 200 and 349/mm^3^ (HR: 1.98; CI: 1.25–3.15; *P* = 0.004) as compared when CD4 count ≥500 cells/µL. The hazard of bacterial pneumonia did not differ between patients with CD4 count between 350 and 499 cells/µL and those with CD4 count ≥500 cells/µL (HR: 0.93; CI: 0.54–1.60; *P* = 0.79). In this final model, the hazard of bacterial pneumonia was also higher among patients with plasma HIV RNA ≥1000 copies/ml (versus <1000 copies/ml) (HR: 1.75; CI: 1.19–2.56; *P* = 0.004), among IDUs versus others (HR: 1.87; CI: 1.27–2.73; *P* = 0.001), among women versus men (HR: 1.56; CI: 1.07–2.27; *P* = 0.02) and among patients aged between 50 and 60 years (HR: 3.53; CI: 1.13–10.98; *P* = 0.03) and those aged ≥60 years (HR: 8.30; CI: 2.37–29.04; *P*<0.001) compared to those aged <30 years (table 2).

## Discussion

Bacterial pneumonia is a major cause of morbidity in HIV-infected patients in the cART era. In our study, the incidence of bacterial pneumonia was 12 per 1000 patient-years, comparing with results reported in previous studies (8 to 20 per 1000 patient-years) [1], [9], [10].

We identified three independent categories of risk factors for bacterial pneumonia: non modifiable risks factors such as age, infection through IDU and gender; HIV infection; and tobacco smoking.

As previously reported, patients infected through IDU had a higher risk of bacterial pneumonia than other patients [10], [18]. This might be explained by a poorer antiretroviral therapy adherence in these patients [19]. Furthermore, daily cannabis consumption, a frequent practice in this category of patient [20], [21], may contribute to this high rate of bacterial pneumonia [22].

HIV-infected women presented a higher risk of bacterial pneumonia than men. This result was observed after adjusting for the main risk factor for bacterial pneumonia. However, we did not adjust our analysis for precarious socio-economic conditions and delayed access to care which are more prevalent in women than men and may increase the risk of bacterial pneumonia [23], [24]. We cannot exclude a higher susceptibility to bacterial pneumonia in HIV-infected women but further research is needed to explore this hypothesis.

We showed that HIV infection increases the risk of bacterial pneumonia through the virus itself and through the related immunodeficiency. As recognized in earlier cohort studies [9], [10], we observed that the risk of bacterial pneumonia was higher in patients with plasma HIV RNA above 1000 copies/ml. One hypothesis is that HIV infection is associated with defects in humoral immunity in the lung, leading to an increase susceptibility to infections [25]. Immunodeficiency is generally reported as the major risk factor of bacterial pneumonia in HIV-infected individuals [1], [18], [26], [27]. In our study, the incidence of bacterial pneumonia dramatically increased in patients with a CD4 count <350 cells/µL compared to those with a CD4 count above this threshold. This emphasizes the need for early HIV diagnosis and initiation of antiretroviral therapy at high levels of CD4 count, in order to preserve cellular immunity [28], [29].

But there are other ways of preventing bacterial pneumonia in HIV-infected patients. Recent studies have reported that pneumococcal vaccination was effective in HIV-infected patients [30], [31]. In our study, the most commonly reported bacterium was *Streptococcus pneumonia*. We did not have any data on the proportion of patients who received a pneumococcal vaccine but these results should emphasize its use in HIV-infected patients. We also reported that 59% of bacterial pneumonia episodes were diagnosed during the period of influenza virus epidemics which is a risk factor for respiratory tract infections [32]. The use of influenza vaccination would also be an important option especially in the context of pandemic situation although data are lacking on its clinical efficacy and safety in HIV-infected patients [33].

Finally, our paper adds original information for the prevention of bacterial pneumonia in HIV-infected patients. Firstly, for the first time in such populations, we showed that after at least one year of tobacco smoking abstinence, the risk of bacterial pneumonia is significantly reduced and compares to the risk observed in never smokers. Secondly, as noted by the absence of interaction between tobacco smoking status and HIV induced immunodeficiency, tobacco smoking cessation is effective in preventing bacterial pneumonia whatever the level of immunodeficiency. These are evidences for promoting tobacco smoking cessation in HIV infected patients. However, we showed that a high proportion of former smokers relapsed during follow-up, even after at least one year of abstinence. This reinforces the need for specific tobacco cessation interventions in such populations to lower the burden of bacterial pneumonia and, more generally, all tobacco-related morbidities.
